# Pediatric tuberculosis-human immunodeficiency virus co-infection in the United Kingdom highlights the need for better therapy monitoring tools: a case report

**DOI:** 10.1186/s13256-017-1222-6

**Published:** 2017-02-26

**Authors:** Dimitrios Evangelopoulos, Elizabeth Whittaker, Isobella Honeyborne, Timothy D. McHugh, Nigel Klein, Delane Shingadia

**Affiliations:** 1 Centre for Clinical Microbiology, University College London, London, NW3 2PF UK; 2 Department of Academic Paediatrics, Imperial College London, London, W2 1PG UK; 3 Department of Paediatric Infectious Diseases, Great Ormond Street Hospital for Children, London, WC1N 3JH UK; 4 Institute of Child Health and Great Ormond Street Hospital for Children, London, WC1N 3JH UK; 5 Present address: Laboratory of Mycobacterial Metabolism and Antibiotic Research, The Francis Crick Institute, London, NW1 1AT UK

**Keywords:** Case report, Tuberculosis treatment monitoring, Molecular bacterial load, Rapid assay, Clinical decisions, Childhood tuberculosis

## Abstract

**Background:**

Tuberculosis is an infection that requires at least 6 months of chemotherapy in order to clear the bacteria from the patient’s lungs. Usually, therapeutic monitoring is dependent on smear microscopy where a decline in acid-fast bacilli is observed. However, this might not be indicative of the actual decline of bacterial load and thus other tools such as culture and molecular assays are required for patient management.

**Case presentation:**

Here, we report the case of a 12-year-old Black African boy co-infected with tuberculosis and human immunodeficiency virus who remained smear culture positive and liquid culture negative for a prolonged period of time following chemotherapy. In order to determine whether there was any live bacteria present in his specimens, we applied the newly developed molecular bacterial load assay that detects the presence of 16S ribosomal ribonucleic acid derived from the bacteria. Using this methodology, we were able to quantify his bacterial load and inform the management of his treatment in order to reduce the disease burden. Following this intervention he went on to make a complete recovery.

**Conclusions:**

This case report highlights the value of improved biomarkers for monitoring the treatment of tuberculosis and the role of molecular assays such as the molecular bacterial load assay applied here. The molecular bacterial load assay detects bacterial ribonucleic acid which corresponds closely with the number of live bacilli as compared with polymerase chain reaction that detects deoxyribonucleic acid and may include dead bacteria.

## Background

Tuberculosis (TB) infection is considered a global health emergency. In 2013, there were an estimated 9 million cases of TB, of which 550,000 (6.1 %) were pediatric [1]. Recent modeling work suggests these pediatric estimates are outdated and only represent 35 % of the true burden [2]. An estimated 13 % of new TB cases were human immunodeficiency virus (HIV) positive and 360,000 of the 1.5 million deaths were in patients who were HIV positive.

TB-HIV co-infection represents a lethal combination: the immunodeficiency induced by HIV leads to progression of TB disease and TB itself decreases the CD4 count and worsens the immunodeficiency caused by HIV in children [3]. Children with HIV infection are 20 times more likely to develop TB and have a six times greater risk of dying from TB than children who do not have HIV infection [3]. Anti-retroviral therapy (ART) has great potential to reduce the risk of TB disease [4]. Diagnosis of TB in patients with HIV is challenging since the presentation may be nonspecific and the combination of clinical features and radiological findings frequently overlap with other common clinical presentations in HIV. In addition, immunological-based assays including the tuberculin skin test (TST) and interferon-gamma release assays may be less sensitive in patients with HIV [5]. Furthermore, the management of TB-HIV co-infection is challenging as concurrent administration of anti-TB therapy (ATT) and ART is associated with significant drug interactions, potentially limiting their efficacy [6].

There is an urgent need for development of new biomarkers that will enable better diagnosis and treatment of TB, particularly in patients with HIV infection. Childhood TB has historically been neglected but recent initiatives are currently examining new therapeutics and novel diagnostic tools specifically for children. One of the key issues for microbiological diagnosis of TB is obtaining timely confirmation of the presence of viable bacteria: culture is slow and can miss specific subpopulations that are viable but non-culturable, whereas mycobacterial deoxyribonucleic acid (DNA) persists beyond the point where there is evidence of viable organisms, making DNA-based assays unsuitable for monitoring the decline of live bacteria in clinical samples [7, 8]. We previously developed a molecular test, the molecular bacterial load (MBL) assay, based on the detection of mycobacterial ribosomal ribonucleic acid (RNA) that detects live mycobacteria independent of those that are dead [9, 10]. This assay has been previously used in retrospective studies for monitoring TB treatment as well as in early bactericidal assays and it is currently implemented in clinical trials by the Pan African Consortium for the Evaluation of Antituberculosis Antibiotics. Here we report a case of pediatric TB-HIV co-infection where the MBL assay, a research tool, was utilized in real time to gain information about the disease progression, inform treatment decisions, and monitor treatment compliance.

## Case presentation

**Fig. 1:**
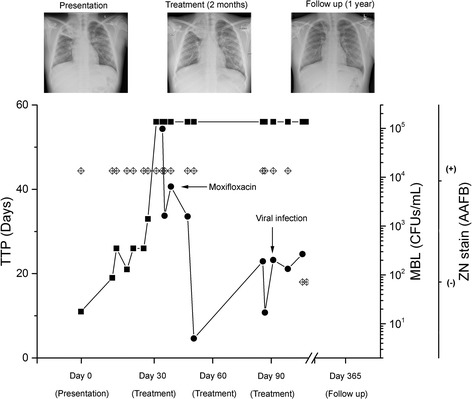
The clinical presentation of the patient over time. Three representative X-ray pictures from patient presentation, during therapy, and follow-up are shown on top of the graph. The time to positivity from culture is reported in days on the left *y*-axis with *black squares*. A time to positivity of 56 is considered to be negative. The molecular bacterial load values are shown on right *y*-axis as *black dots*. Smear microscopy is referred to as acid-fast bacilli positive or acid-fast bacilli negative for positive or negative samples respectively and is shown on the graph as *diamonds*. *AAFB+* acid-fast bacilli positive, *AAFB–* acid-fast bacilli negative, *CFU* colony-forming unit, *MBL* molecular bacterial load, *TTP* time to positivity, *ZN* Ziehl–Neelsen

We present the case of a 12-year-old Black African boy with perinatally acquired HIV infection and pulmonary TB. He was born in the United Kingdom (UK) and did not receive the Bacillus Calmette–Guérin (BCG) vaccine. He remained clinically well and off ART until 2013 when he developed recurrent episodes of shingles. At this time, his CD4 count fell below 500 cells/mm^3^ for the first time. He commenced ART with Kivexa® (abacavir/lamivudine), darunavir, and ritonavir in March 2014. In July 2014, he presented to his local accident and emergency department (A&E) with a cough associated with hemoptysis. At this time he reported a 6-month history of non-productive cough, loss of appetite, and weight loss. He denied fever and night sweats. He had travelled to Kenya in 2012 with his family, but had no confirmed contact with individuals with active TB. A sputum sample was found to have acid-fast bacilli (AFB) on microscopy. A chest X-ray and computed tomography (CT) scan of his thorax were indicative of pulmonary TB infection (Fig. 1). He was transferred to Great Ormond Street Hospital for Sick Children, National Health Service (NHS) Trust, London, UK for ongoing care. He was commenced on ATT with isoniazid, rifabutin, ethambutol, and pyrazinamide as per local guidelines. He had no further episodes of hemoptysis and following review by the thoracic surgeons and radiologists, it was agreed he did not require intervention at that stage (Fig. 1). The AFB were confirmed to be *Mycobacterium tuberculosis* complex and found to be fully drug sensitive via drug susceptibility testing.

Despite being on an appropriate treatment regimen, his sputum remained smear positive. In view of this, repeat resistance testing was performed that did not show evidence of resistance to first-line therapy. For this reason, an MBL assay was performed on serial sputum samples in order to examine the status of the bacilli. This assay demonstrated that despite a month of effective therapy there were still detectable numbers of live bacteria in the sputum samples (Fig. 1). On the basis of these results, his ATT was modified to moxifloxacin, isoniazid, pyrazinamide, and ethambutol. He was discharged home on this regimen under video observed therapy (VOT) and reviewed regularly by the Pediatric TB service to ensure compliance with treatment. Serial sputum samples tested using the MBL assay over the next 2 weeks demonstrated declining numbers of live bacteria, despite ongoing sputum smear positivity (Fig. 1).

He remained clinically well until an out-patient clinic review 4 weeks later. At this time, he had an upper respiratory tract infection and a new onset cough. A sputum sample at this time was smear positive. He was readmitted and treated with oral co-amoxiclav for a presumed intercurrent infection. Nasopharyngeal aspirate and sputum sample testing did not reveal a virological or bacteriological cause for this. A repeat chest X-ray showed radiological improvement (Fig. 1). A further sputum sample sent 3 days later was also smear positive. Both of these samples were also sent for MBL assay in addition to mycobacterial culture via the BACTEC MGIT 960 System (Becton Dickinson) and resistance testing. The MBL assay confirmed a very low bacterial load, ~200 colony-forming units (CFUs), whereas culture and resistance testing was negative. Clinically he continued to improve and all further samples were smear and culture negative (Fig. 1). He has had no further sputum smear or culture positive results and completed a 9-month treatment course without further incident.

## Conclusions

In this case of prolonged sputum smear positivity, the MBL assay was extremely useful both for informing the decision to change from rifabutin to moxifloxacin and for following this to confirm mycobacterial response to the appropriate anti-tuberculous treatment. In addition, when he presented with renewed sputum smear positivity, presumably due to mobilization of dead mycobacteria secondary to a viral-induced cough, the MBL assay provided reassurance that there was a very low bacterial load in his sputum samples.

Our patient responded well both clinically and with decreasing levels of live bacteria as measured by the MBL assay to a switch in treatment from rifabutin to moxifloxacin. Last generation fluoroquinolones have anti-TB bactericidal activity that is comparable with rifamycins and data from adult patients suggest that this treatment is efficacious [11]. There are no clinical trials on the use of these drugs in children, although a recent pharmacokinetic and safety study for multidrug-resistant TB in children supports their use [12]; however, further studies are required before they can be routinely recommended for drug-sensitive TB.

It is clear that effective treatment monitoring can be utilized for TB infections and a rapid biomarker such as the MBL assay, which enables the enumeration of live mycobacteria in sputum, is vital for appropriate patient management and to avoid development of resistance. Since smear microscopy cannot differentiate between live and dead bacilli and some populations of *M. tuberculosis* might not be recovered using microbiological-based culture, a molecular tool that utilizes bacterial RNA for its detection can identify accurately the presence of live bacteria in clinical samples [13]. Our patient had a complete recovery; however, his last MBL tests constantly exhibited values of around 200 CFUs (Fig. 1). The significance of these low numbers of bacteria in the clinical samples and their potential relevance for causing further disease progression or relapse is unclear. One hypothesis is that the immune system can perhaps effectively contain these low numbers of bacilli without any clinical symptoms, but further work is required.

## Abbreviations

A&E: Accident and emergency department
AFB: Acid-fast bacilli
ART: Anti-retroviral therapy
ATT: Anti-tuberculosis therapy
BCG: Bacillus Calmette–Guérin
CFU: Colony-forming unit
CT: Computed tomography
HIV: Human immunodeficiency virus
MBL: Molecular bacterial load
NHS: National Health Service
TB: Tuberculosis
TST: Tuberculin skin test
VOT: Video observed therapy
